# 689 Documentation of Occupational Therapy and Physical Therapy Dosage for Children with Burns

**DOI:** 10.1093/jbcr/iraf019.318

**Published:** 2025-04-01

**Authors:** Taylor Iske, Sara O’Rourke, Renata Fabia, Rajan Thakkar, Dana Schwartz, Ben Reader

**Affiliations:** Nationwide Children’s Hospital; Nationwide Children’s Hospital; Nationwide Children’s Hospital; Nationwide Children’s Hospital; Nationwide Children’s Hospital; Nationwide Children’s Hospital

## Abstract

**Introduction:**

Burns are the fifth most common non-fatal pediatric injury worldwide, and commonly require therapeutic input to optimize outcomes. Rehabilitation interventions in adults with burns can decrease hospital length of stay and improve outcomes, however similar evidence is lacking for pediatric burn patients. To optimize delivery of rehabilitation services, it is first important to accurately and objectively capture the dose of occupational therapy (OT) and physical therapy (PT) interventions. Therefore, we aimed to describe the application of a newly developed documentation style for OT and PT to hospitalized children with burn injuries and explore its potential for improving outcomes.

**Methods:**

A retrospective cohort study from 2022-2023 examined the demographics and OT/PT visit-level data using the electronic health record (EHR) of children (0-21 years) admitted with thermal injuries to a single ABA-verified pediatric burn center. OT/PT visit data was automatically extracted from a newly developed documentation template that includes discrete fields to describe the frequency (visits per week), length of session (time), and type/s of therapeutic interventions provided. Data were summarized using descriptive statistics.

**Results:**

A total of 51 patients received OT (n=33) and PT (n=34). Most children were White (41.2%), non-Hispanic/Latino (94.1%), male (60.8%), and were burned due to a scald injury (56.9%). Frequency of sessions was commonly prescribed at 6x/week (OT: 60.6%; PT: 79.4%). The mean treatment time for OT sessions was 38 (SD: 19) minutes and 34 (SD: 17) minutes for PT sessions. In 146 sessions, the most utilized interventions for OT (n=146 sessions) were active range of motion (ROM; 52.4%), task specific practice (30.6%) and fine motor/dexterity (27.9%). Most common interventions for PT were active ROM (66.2%), positioning (58.8%), functional strengthening (48.0%) and scar management (48.0%).

**Conclusions:**

This newly developed documentation style generates EHR data that not only reliably records specifics of each therapy visit, but is also easily extracted to correlate with patient outcome data. In this cohort, children with acute burns received OT/PT services at a high frequency, for a varied duration, and predominately focused on active ROM exercises.

**Applicability of Research to Practice:**

This novel documentation style enables tracking of therapy dosage, providing valuable insights into current practices. This documentation style aids in the identification of gaps in care and allows for comparison with the best available evidence. By standardizing documentation and monitoring dosage, this may improve the quality of rehabilitation interventions and support future research aimed at optimizing pediatric burn rehabilitation.

**Funding for the Study:**

N/A

